# Magnetic Resonance Imaging of Water Content and Flow Processes in Natural Soils by Pulse Sequences with Ultrashort Detection

**DOI:** 10.3390/molecules26175130

**Published:** 2021-08-24

**Authors:** Sabina Haber-Pohlmeier, David Caterina, Bernhard Blümich, Andreas Pohlmeier

**Affiliations:** 1 Institute for Modelling Hydraulic and Environmental Systems, University of Stuttgart, Pfaffenwaldring 61, D-70569 Stuttgart, Germany; sabina.haber-pohlmeier@iws.uni-stuttgart.de; 2 Institute for Bio- and Geosciences Agrosphere (IBG-3), Research Center Jülich, D-52425 Jülich, Germany; David.Caterina@uliege.be; 3 Département UEE, Faculté des Sciences Appliquées, Université de Liège, B-4000 Liège, Belgium; 4 Institute for Technical and Macromolecular Chemistry, RWTH Aachen University, Worringer Weg 2, D-52074 Aachen, Germany; bluemich@itmc.rwth-aachen.de

**Keywords:** magnetic resonance imaging, natural soil material, fast relaxation times, water content, water flow

## Abstract

Magnetic resonance imaging is a valuable tool for three-dimensional mapping of soil water processes due to its sensitivity to the substance of interest: water. Since conventional gradient- or spin-echo based pulse sequences do not detect rapidly relaxing fractions of water in natural porous media with transverse relaxation times in the millisecond range, pulse sequences with ultrafast detection open a way out. In this work, we compare a spin-echo multislice pulse sequence with ultrashort (UTE) and zero-TE (ZTE) sequences for their suitability to map water content and its changes in 3D in natural soil materials. Longitudinal and transverse relaxation times were found in the ranges around 80 ms and 1 to 50 ms, respectively, so that the spin echo sequence misses larger fractions of water. In contrast, ZTE and UTE could detect all water, if the excitation and detection bandwidths were set sufficiently broad. More precisely, with ZTE we could map water contents down to 0.1 cm^3^/cm^3^. Finally, we employed ZTE to monitor the development of film flow in a natural soil core with high temporal resolution. This opens the route for further quantitative imaging of soil water processes.

## 1. Introduction

Water content and flow in soils belong to the most important processes controlling plant growth and crop yield. They take place on different scales ranging from the field scale down to the pore scale. On a coarse-grained scale the pore system is a continuum, which one may classify in five categories, of which the three most important ones are made up from macropores with voids >75 μm, such as wormholes or dead root holes, mesopores with voids between 75 and 30 μm, and micropores, smaller than 30 μm [1]. The water content of the meso- and micropores in the soil matrix is controlled by capillary suction, whereas macropores are mostly empty with thin water films on the walls. Water flows predominantly through the pore system in the soil matrix, i.e., in the micro- and mesopores. Under certain conditions, such as re-wetting after severe desiccation combined with the formation of cracks or high irrigation rates, preferential flow through the macropore system can significantly contribute to the total water flow. This may take place on each scale between the macropores (10^−3^ m), the core scale (10^−1^ m) up to the pedon and field [2]. While the larger scales >10^0^ m are conveniently investigated by, e.g., TDR probe arrays, surface NMR [3] or geophysical methods [4], there is need for high resolution, non-invasive imaging addressing the core and soil aggregate scales. 

**Figure 1:**
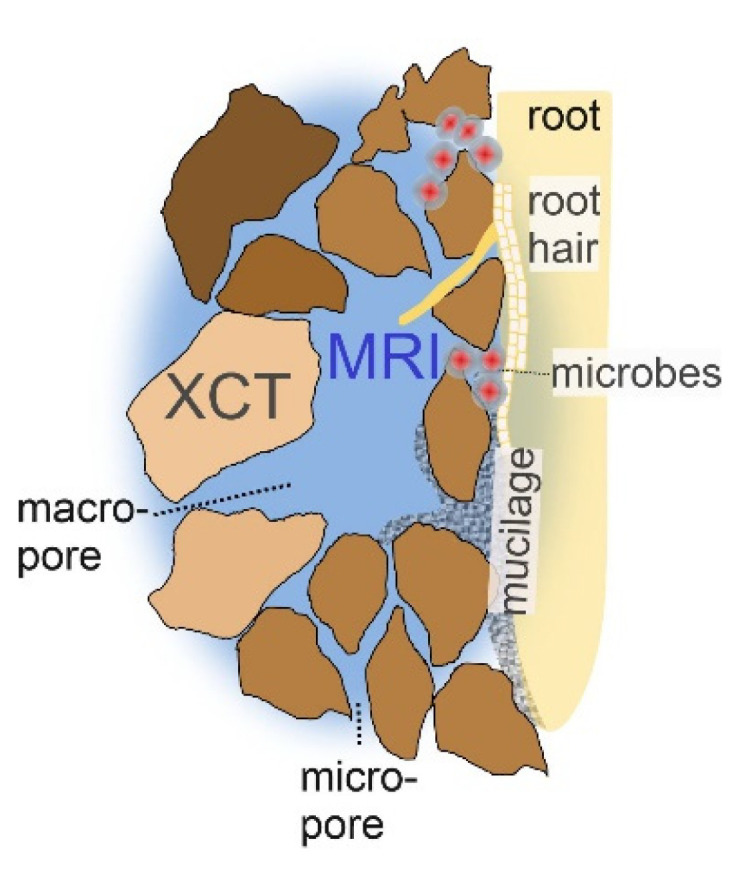
Sketch of the pore system and the domains addressed by different non-invasive imaging methods. X-ray computed microtomography (XCT) is sensitive to the solid soil structure and magnetic resonance imaging (MRI) is sensitive to the fluid in the pore system and its dynamics.

Three-dimensional, non-invasive imaging techniques are promising tools to improve our understanding of the interplay between soil structure, water content and flow processes (Figure 1). These methods include X-ray CT, an excellent tool for studying the microstructure of soils, i.e., the spatial distribution of minerals and pore networks. Sample and detector sizes control the resolution, where the current limit is about one micrometer for 1 mm wide samples [5,6]. While the contrast in XCT images relies on the density difference between soil particles and voids, magnetic resonance imaging (MRI) and its special subdiscipline magnetic resonance microscopy (MRM) directly probe the local dynamics of the molecule of interest in the pore void: water [7,8,9,10]. This makes MRI especially convenient for the investigation of stationary and mobile water in the soil pore system (Figure 1). 

The NMR principle is that certain atomic nuclei possess a quantum-mechanical property called spin, which is linked to a nuclear magnetic moment and interacts with the external magnetic field ***B***_0_. Many spins form an ensemble with a macroscopic magnetization aligned with the direction of ***B***_0_ in equilibrium. This equilibrium can be disturbed by the application of a radio frequency pulse if its frequency matches the Larmor-frequency of the spin system, *ν*_0_ = *γ B*_0_/2π, where *γ* is the gyromagnetic ratio of the nucleus under consideration. It is, in most cases, the hydrogen atom ^1^H, which is abundant in water or hydrocarbons. After excitation, the nuclear magnetization induces a current in the surrounding *rf* coil, which produces the NMR-signal. Further excitation pulses, frequently combined with magnetic field gradient pulses can modify the signal—for instance, to create one or more echoes. In parallel, the equilibrium is re-established by characteristic relaxation processes, denoted as *T*_1_ and *T*_2_ relaxation, sensitive to the physical and chemical environments. The observable signal relies on the interplay between magnetic and dynamic (rotational and translational diffusion, flow) properties of the actual system and method-specific, adjustable parameters. The spatial coordinates of an image are encoded in MRI by switching additional magnetic field gradients before or during signal acquisition. The sequential application of all pulses is termed the MRI pulse sequence. Eventually, Fourier transformation of a time series of NMR signals obtained with systematic variation of the pulsed magnetic field gradients yields the 2D or 3D image. The versatility of the information of MRI is due to the fact that the signal intensity of the individual image pixels, i.e., the contrast, is controlled by factors such as the volumetric water content, the NMR relaxation times *T*_1_ and *T*_2_, diffusion coefficients, and flow velocity, which allows tuning the MRI pulse sequence to be sensitive to certain of these parameters. For instance, the so-called spin-echo multislice sequence (SEMS) is sensitive to the water content if the shortest *T*_2_ relaxation time in the sample is significantly longer than the echo time *t*_E_. On the other hand, if *T*_2_ becomes equal to or shorter than *t*_E_, the pixel intensity is weighted by the local relaxation properties. This has consequences for MRI of natural soil material since soils are natural porous media with a broad distribution of pore sizes and significant fractions of organic matter, clay minerals and paramagnetic ions such as iron or manganese, which greatly impact the soil relaxation times. Thus, the pixel intensity of a SEMS sequence can significantly decrease for such systems with short *T*_2_ since *t*_E_ cannot be minimized beyond certain limits, and water content is not reliably mapped [11,12].

The limitation caused by fast *T*_2_ relaxation times has driven the development of a family of different pulse sequences with ultrashort detection times. They have in common that they avoid the time-consuming creation of a spin-echo so that the image intensity is given by the free induction decay (FID), which decays with the relaxation time *T*_2_*, and the longitudinal relaxation time *T*_1_. One may differentiate between two classes. Single point imaging methods (SPI, SPRITE) are purely phase encoded and thus do not suffer from susceptibility artefacts [13,14], but require relative long measurement times, especially for 3D imaging. On the other hand, sequences such as ultrashort echo time imaging (UTE) [15], sweep imaging with Fourier transformation (SWIFT) [16], and zero echo time (ZTE) [17,18] allow very short measurement times of some minutes by using frequency encoding of the image dimensions in two or three directions. Their disadvantage is their sensitivity for artefacts caused by internal magnetic field gradients occurring at interfaces between structures with significantly different magnetic susceptibilities. Strategies to address this are the use of extremely short and broadband *rf*-pulses and short acquisition times. Besides medical applications such as the imaging of bones, tendons, or certain parts of the brain [19,20], these methods are of high convenience in the geo- and material sciences. Examples are fluid content imaging using SPRITE in rock cores [21,22,23] or mortar [24]. SPRITE is also especially convenient for rapid moisture profiling combined with relaxation time analysis in soil cores [25]. The relative long measuring times motivated other groups to use 3D ZTE instead for imaging fractures in rocks [26] or moisture ingress in rock cores [27,28] where the interpretation is strengthened by correlation with X-ray CT images of the solid matrix. Since ZTE detects also signal from plastic cuvette materials, difference images of the sample and the empty plastic sample holder can be computed so that the scattering of intensity from the plastic holder into the sample was minimized [27].

To overcome the issue caused by the rapid transverse relaxation of water in natural soil materials in the millisecond range [11,12], the objective of this study was to explore the usefulness and applicability of the ultrashort pulse sequences UTE and ZTE for mapping water contents in natural soils. First, we started with the investigation of the relaxation behavior of a selected soil material followed by the determination of optimal pulse sequence parameters for ZTE and UTE with special focus on the interplay between the excitation and detection of pulse bandwidths, acquisition time and flip angles. We continued with the question, if water content can be mapped quantitatively with these methods for different degrees of unsaturation. The final example is the application of ZTE in a case study of water ingress into a natural soil core to answer the question if it is suitable to monitor rapid transient changes of water content occurring in macropore flow.

## 2. Results

### 2.1. Relaxometric Imaging, Sample S1

**Figure 2:**
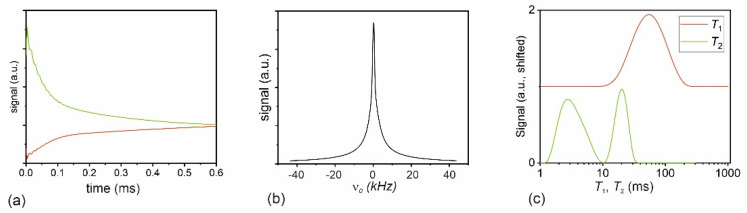
Saturated sandy loam sample S1. (**a**) FID with real and imaginary part in green and red. (**b**) NMR magnitude spectrum. The linewidth is 2.09 kHz corresponding to *T*_2_* = 0.15 ms. (**c**) Relaxation spectra from a selected region of interest (ROI) centered in one cuvette. The *T*_2_ spectrum was obtained by inverse Laplace transformation of the average intensity of a ROI mapped by a multislice multiecho imaging pulse sequence using an echo time of 1.6 ms and 32 echoes. The *T*_1_ spectrum was obtained from a series of images measured by a multislice single echo imaging pulse sequence with IR preparation with inversion times between 4.2 ms and 300 ms. The average *T*_1_ is 50 ms, *T*_2_ exhibits a fast mode of 3 ms and a slower mode at 20 ms.

Soil materials frequently possess short transverse and longitudinal relaxation times due to their considerable content of paramagnetic ions and clay minerals [11,12]. Therefore, the relaxation properties of the sample need to be determined before acquiring images for an optimal setup of the imaging pulse sequence parameters. As an example, Figure 2a,b show the FID of the saturated sandy loam sample S1 (Table 1) and the corresponding spectrum. The FID is short with an average *T*_2_* of about 0.15 ms, and the corresponding spectrum has a width at half height of 2.09 kHz whereby the line is not a single Lorentzian. This makes imaging pulse sequences using the FID such as ZTE or UTE prone for *T*_2_* blurring so that the acquisition time should be kept as short as possible. Furthermore, gradient echo sequences, frequently used in biomedical imaging, will deliver inferior results, since most of the signal will have decayed at the time the echo is created. Another family of imaging pulse sequences relies on the Hahn echo, whose intensity is controlled by the transverse relaxation time *T*_2_. The transverse relaxation spectrum in Figure 2c shows a very fast component at 3 ms associated with clay-bound and micropore water and a slower component at 20 ms caused by immobile water in the capillary pores. Larger fractions of free water in the macropores were not visible since they were drained under the given conditions. The high fraction of fast relaxing water indicates a considerable loss of image intensity also in spin-echo sequences since the echo time cannot be reduced below a technical limit. The average longitudinal relaxation time *T*_1_ was 50 ms so that the Ernst angle is 28° for a repetition time of 6 ms. Therefore, if the adjusted flip angle is significantly smaller, no interference of saturation on the signal is expected and the signal intensity will be proportional to the volumetric water content. 

### 2.2. Comparison of MSME and ZTE, Sample S1

**Figure 3:**
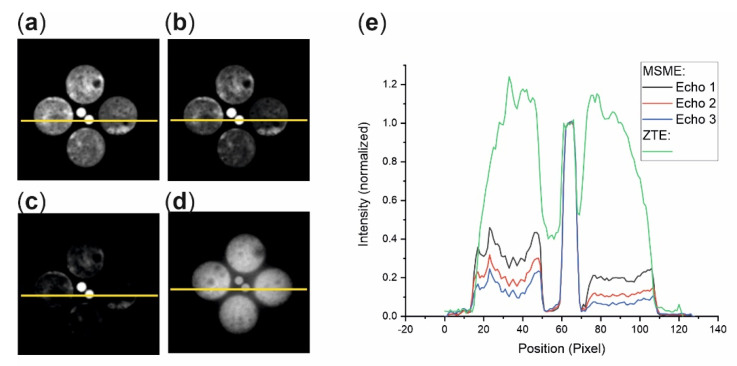
Comparison of MSME and ZTE images of sample S1. The marker tubes in the middle contained 33% water. (**a**–**c**) MSME images of a central axial slice through S1 of echoes 1, 2, and 3. Echo time *t*_E_ = 1.6 ms, slice thickness = 2 mm, matrix size 128^2^, FOV: 7 × 7 cm^2^. (**d**) ZTE image of an axial slice, FOV 7 × 7 × 9 cm^3^, matrix size = 128^3^ resulting in an in-plane resolution of 0.55 mm and a slice thickness of 0.7 mm. (**e**) Intensity profiles along the horizontal yellow lines, normalized to the intensity of the reference tube containing 0.33 vol-% water.

Following the initial relaxation analysis, the effects of relaxation on images acquired with different methods are checked. The MSME images of sample S1 yielded an image with sufficient intensity only for the first echo at *t*_E_ = 1.6 ms, whereas the intensities of the subsequent echoes decreased significantly (Figure 3a–c). For elucidation, Figure 3e depicts the intensity profiles of some echo images along the yellow line. Even the intensity of the first echo image from the soil was not proportional to the volumetric water content due to the loss of immobile water associated with clay and micropores as revealed by comparing the image intensities with the intensities of water in the marker tubes. The latter remained approximately constant and reflected the volumetric water content of 0.33 cm^3^/cm^3^. In contrast, the ZTE image (Figure 3d) represented the correct water content, as can be seen by the green profile in Figure 3e. One also notes a slight decay of the ZTE intensity with increasing distance from the center of the field of view. It was caused by the spatial heterogeneity of the *rf*-irradiation intensity, which had to be compensated for by normalizing the ZTE images to an image of a homogeneous reference sample (cf. next section).

### 2.3. Comparison ZTE and UTE Sequences, Sample S2

**Figure 4:**
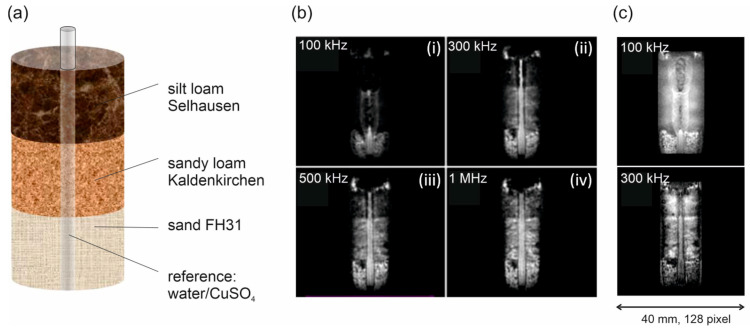
Imaging a composite soil sample S2 with UTE and ZTE. (**a**) Sketch of the phantom. (**b**) UTE images of coronal cross-sections showing the three soil materials and the marker tube in the center. Matrix size and resolution are 128^3^ and isotropic 0.31 mm, respectively. Increasing the receiver bandwidth (reduction of acquisition time) from 0.1, 0.3, 0.5, to 1.0 MHz decreased *T*_2_* blurring artefacts especially for the silt loam soil material. (**c**) ZTE Images: Increasing the receiver bandwidth from 100 to 300 kHz reduced the *T*_2_* blurring and improved the image quality.

The rapid *T*_2_* decay causes not only blurring but makes the ultrashort pulse sequence prone for artefacts if the acquisition and excitation bandwidths are too small. This becomes clear when investigating the composite sample S2 (Table 1) with soil materials of different texture, iron content and *T*_2_* (Figure 4a). In the top-left image of Figure 4b (i) the fine-textured silt-loam soil is practically invisible, as well as the marker tube in the top compartment. The marker tube becomes more visible in the central compartment filled with the medium textured sandy loam material, although the intensity is distributed laterally due to the susceptibility artefact. The susceptibility difference between soil and marker tube creates local magnetic field gradients, which shift the local resonance frequency and lead to shifted intensities in frequency-encoded images which UTE and ZTE are. 

The marker tube is represented correctly only in the sand compartment. The situation improves when the acquisition bandwidths are increased so that the space-encoding gradients become stronger and exceed the internal gradients (Figure 4b (ii) to (iv)). The same is true for the ZTE sequence (Figure 4c). Using a small bandwidth of 100 kHz results in strong artefacts, which are considerably reduced by increasing the bandwidth to 300 kHz. As an interim conclusion both sequences are convenient for soil systems; however, we performed the following experiments in this paper with ZTE. 

### 2.4. Check for Volumetric Water Content, Samples S3 and R1

**Figure 5:**
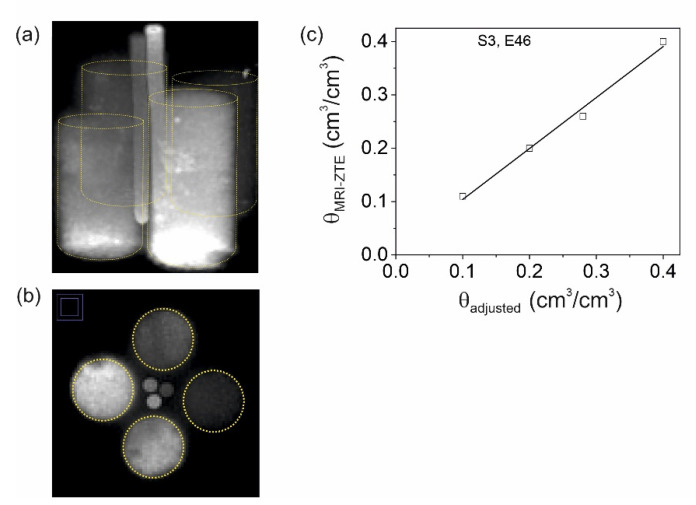
Quantification of water content in sandy loam by means of MRI-ZTE, sample S3. (**a**) The maximum intensity projection shows the four cuvettes with volumetric water contents of 0.10, 0.19, 0.28, and 0.40 cm^3^/cm^3^. The raw images were normalized firstly on the homogeneity phantom R1 to compensate for radial signal-intensity inhomogeneities and secondly on the signal intensity from the reference cuvettes visible in the center with known volumetric water contents. The yellow wireframes indicate the positions of the cuvettes. (**b**) Axial cross section through the ZTE image. (**c**) Water content obtained from MRI-ZTE versus volumetric water content from sample weights.

To check the expected linearity between the MRI-ZTE signal with volumetric water content, we scanned in the next step a bundle of four soil-containing cuvettes with different water contents (Sample S3, Table 1). It turned out that the homogeneity of the rf-field was too low so that the intensities near the inner wall of the resonator and more than 2 cm below and above its center decreased, and the image intensities had to be corrected accordingly. For that purpose, we additionally scanned the homogeneity phantom sample R1 with identical pulse-sequence parameters. The 3D ZTE image of the bundle was normalized to this 3D reference image (Figure 5a,b). The slight blurring which is visible in the axial cross sections in Figure 5b is due to the rapid *T*_2_* decay during the acquisition time of 0.213 ms. This means that the signal of the data points acquired in the outer ***k***-space, which determine the resolution, is attenuated with respect to the signal in the center. In contrast to the soil cuvettes, the marker tubes yielded sharp images since *T*_2_* of the CuSO_4_ solutions is considerably longer (*T*_2_* = 1 ms). Finally, we divided the normalized image by the intensity of one of the marker tubes in the center with the known water/D_2_O ratio yielding the volumetric water content map of the bundle. The results for a selected central slice are plotted in Figure 5c, confirming the expected linearity between the given and the MRI-ZTE water content. 

### 2.5. Infiltration into a Natural Soil Core, Sample S4 

**Figure 6:**
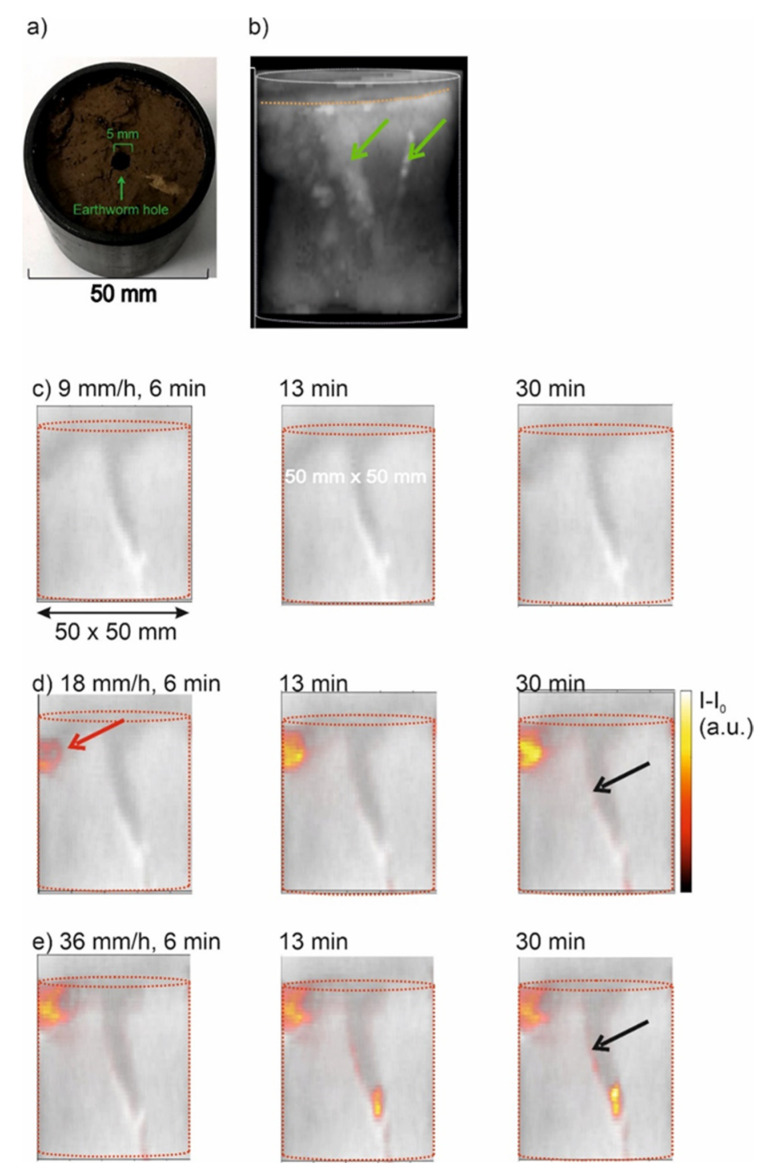
Imaging flow in a natural soil core during irrigation. (**a**) Setup. (**b**) ZTE image of the natural core (maximum intensity projection) with no irrigation. Matrix size 128^3^, FOV: 70 × 70 × 90 mm. The dotted orange line indicates the soil surface, the green arrows point to the large wormhole and a smaller macropore, probably caused by a degraded root. (**c**–**e**) Central vertical slice with different irrigation rates (top to bottom) and time-points after start (left to right). Shown are difference images at the given time-points minus t = 0. The red arrow indicates ponding water near the left edge. The black arrows indicate film flow at the walls of the large wormhole.

After the optimized parameters for ZTE had been determined, we applied the method on an infiltration experiment in a natural soil core (Table 1). The core (Figure 6a) was initially saturated from the bottom and scanned. The central large wormhole and a neighboring macropore, probably caused be a degraded root, are clearly visible in Figure 6b (green arrows). The soil surface is uneven, and declined to the left side (dotted orange line in Figure 6b). 

After some time of equilibration, the first irrigation period of one hour started, monitored by frequent MRI-ZTE scans. During this low irrigation rate of 9 mm/h the water content remains almost constant since the difference images did not change even after 30 min (Figure 6c). The situation changed when doubling of the irrigation rate (Figure 6d). After 6 min ponding water accumulated on the top left corner of the soil column with a minor degree of penetration into the topmost soil area. After 13 min the ponding increased giving rise to film flow in the large wormhole. This effect was even more pronounced after 30 min—see the black arrow in Figure 6d, right panel. When the irrigation rate was further increased to 36 mm/h, the ponding was more pronounced, as well as the film flow inside the wormhole (Figure 6e). After 13 min further ponding at the lower end of the wormhole was noticed, which further intensified after 30 min. Summarizing the observations from all images, two points become clear: (1) Steady states were established after a few minutes—this means that the water content patterns did not change significantly after 13 min. Not shown, but worth mentioning is that the reverse process, the desiccation after stopping the irrigation took place as quickly as the irrigation, i.e., a steady state was established in the same period as after starting the irrigation; (2) The onset of film flow inside the wormhole at an irrigation rate *I* > 18 mm/s proves that at this point the irrigation rate exceeded the hydraulic conductivity of the soil matrix. The hydraulic conductivity was sufficiently high below this critical rate to transport all incoming water through the soil matrix so that no preferential flow was visible. Only if the irrigation rate exceeded this value did the macropore flow contribute to a significant fraction of the overall water flux. 

## 3. Discussion

Natural soil cores exhibit fast NMR relaxation processes characterized by the longitudinal relaxation time *T*_1_ and the transverse relaxation time *T*_2_. In terms of NMR imaging the transverse relaxation time is the most critical parameter since values on the order of some ms significantly reduce the signal intensity of conventional, echo-based MRI images of the water in the soil matrix. The relaxation time spectrum of the examined sandy loam exhibited short *T*_2_ components in the range of 3 ms and an average value of *T*_2_* = 0.15 ms, which fit well in the range reported for many other soil materials by Hall et al. [11]. One should further keep in mind that *T*_2_ depends strongly on the echo time at higher field strengths due to a significant contribution of diffusional attenuation of the spin-echo by the Bloch–Torrey term. Therefore, a further reduction in *T*_2_ is expected with increasing echo-time. Summarizing, larger water fractions might become undetectable, and spin-echo based pulse sequences are only convenient for the detection of preferential pathways in macropores or fractures [29,30]. A way out for quantitative water content mapping are pulse sequences with ultrashort detection time, which probe the FID directly a few microseconds after the excitation pulse. Although several methods, such as SPI, SPRITE, or UTE are suitable, we focused on ZTE due to its robust implementation and short total scan time.

Pulse sequences with ultrashort detection time are inherently *T*_1_ weighted since they employ short repetition times to allow multiple data acquisition periods for improving the S/N ratio. Therefore, the flip angle should be significantly smaller than the Ernst angle for the given ratio of repetition time *t*_R_ and average longitudinal relaxation time *T*_1_ to minimize *T*_1_-weighting. For example, the Ernst angle of the sandy loam soil samples S1, S2, and S3 was 28° for a repetition time of 6 ms and a mean *T*_1_ of 50 ms. Thus we employed flip angles in the range of 3° in the UTE and ZTE protocols to assure the proportionality between pixel signal and volumetric water content.

The sample holder and probe head are frequently made of hydrogen atom-containing plastic materials. These contribute to very rapidly decaying FIDs and thus remain visible with ultrashort detection pulse sequences. In fact, such materials lead to diffuse, heterogeneous image backgrounds that should be corrected. A first correction is a sufficiently large field of view that covers at least a part of the probehead, since the rf-coil always excites these probehead components [18]. Secondly, one can normalize the image of interest on a homogeneous phantom, for instance a water/D_2_O mixture in a PTFE cuvette, which has been recorded with identical parameter settings. This strategy was used to validate the quantitative water content imaging in Figure 5. The linearity in Figure 5c between adjusted and MRI measured water content confirms the suitability of ZTE for soil material. Alternatively, if changes in the water content are of interest, one can also calculate difference images of the state at a given point in time and a reference state, which has been successfully applied for water infiltration and desiccation in rock cores [27,28]. We used this procedure in our work to monitor film flow formation as a function of irrigation rate in a core of natural soil (Figure 6). This example shows that it is possible to follow such transient processes with high temporal resolution. 

ZTE and UTE are pure frequency encoding sequences, which makes them prone for artefacts by adjacent pixels with a high contrast in *T*_2_*. An example is shown in Figure 4 where the vertical water-filled NMR tube became essentially invisible when the acquisition bandwidth was set too low. A way out are stronger read-out gradients accompanied by shorter dwell time and acquisition time, which reduces *T*_2_* blurring. Likewise noteworthy is that the excitation in the ZTE sequence takes place in the presence of strong gradients by short rf-pulses with high power (hard or block pulses). Therefore, one should use a wide excitation bandwidth (here 1.28 MHz) so that the approximate linear range of the central lobe covers the entire field of view.

An alternative to ZTE or UTE is the use of pure phase encoding sequences, i.e., the family of single point imaging pulse sequences. They were originally developed for imaging solids, but also proved very useful for imaging rapidly relaxing fluids in natural porous media [13,20,31,32]. They do not suffer from the artefacts addressed above but demand a long overall measuring time. For instance, using a repetition time of *t*_R_ = 6 ms and a matrix size of 64^3^ pixels the total time for a single scan is about ½ h. While this is acceptable for samples in stationary state, for one-dimensional profiling, and 2D imaging [25,33], it might be too long for monitoring rapid transient processes such as film flows. However, SPI in combination with compressed sensing could be also a useful tool [34].

## 4. Materials and Methods

### 4.1. MRI Methods and Image Processing

**Figure 7:**
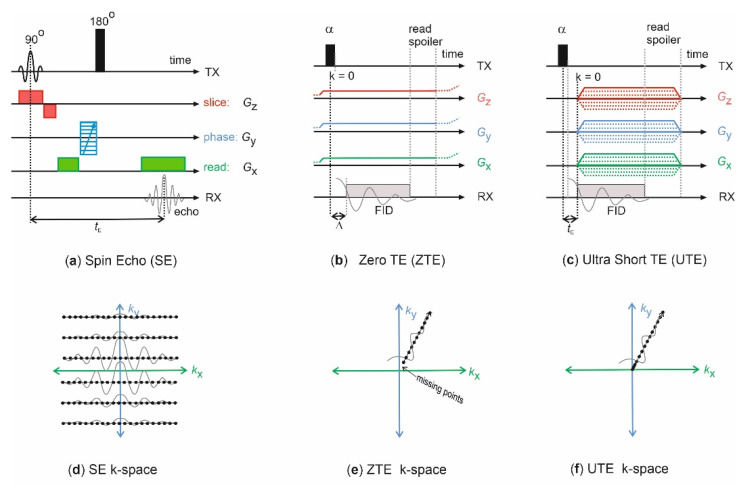
Simplified MRI pulse sequence diagrams. (**a**) Spin-echo (SE) sequence, also termed multislice multiecho sequence (MSME), if more than one echo more recorded per excitation and phase-encoding step. A combination of the 90° rf pulse with the slice gradient *G*_z_ excites an individual slice, the 180° *rf* pulse refocuses the dephased magnetization in the *xy*-plane and creates an echo after echo time *t*_E_, which is read-out in presence of the read gradient *G*_x_ and stored. The phase gradient *G*_y_ encodes the 3rd dimension. Thus, the corresponding ***k***-space (**d**) is filled in Cartesian coordinates. (**b**) In the ZTE sequence a non-slice selective hard *rf*-pulse excites the spin system with a flip angle *α* after the three spatially encoding gradients, *G*_x_ *, G*_y_, and *G*_z_ have been switched on. Instead of an echo, the FID is monitored by *n*_acq_ complex data points after the dead time *Δ*. Different combinations of the gradients separated by the spoiling time achieve spatial encoding. (**e**) The ***k***-space is filled radially, shown is one exemplary projection line. (**c**) In the UTE sequence a non-slice selective hard *rf*-pulse excites the spin system with a flip angle *α* before the three spatially encoding gradients, *G*_x_ *, G*_y_, and *G*_z_ are switched on. Like in ZTE, the FID is monitored and stored, and space is encoded by different combinations of the gradients. (**f**) The ***k***-space is filled radially.

Most samples (with the exception of sample S2) were scanned using a Bruker superwidebore scanner (SWB) at *B*_0_ = 4.7 T, operated by an Avance III console and controlled by Paravision 6 software (Bruker Microimaging, Rheinstetten, Germany). The gradient system had a maximum strength of 0.6 T/m, and we used a ^1^H probehead with 66 mm internal diameter. We used the following pulse sequences provided by the manufacturer: multislice multiecho (MSME), zero-TE (ZTE) and ultrashort TE (UTE) with the parameters specified below. Figure 7 depicts schematically the pulse sequence diagrams. Additionally, sample S2 was scanned with UTE and ZTE in the applications lab of Bruker in Rheinstetten using a WB scanner at *B*_0_ = 7T. The ZTE and UTE image were reconstructed by re-gridding to Cartesian coordinates of 128^3^ points without further filtering or zero-filling by Paravision, and finally displayed by Fiji [31].

### 4.2. Sample S1

We assembled a bundle of four quartzglass cuvettes with 20 mm inner diameter, filled with repacked soil material from Kaldenkirchen. Its textural composition is summarized in Table 1. Iron content was 0.25% [32]. The bulk density was 1.55 g/cm^3^ corresponding to a porosity of 0.41 cm^3^/cm^3^. The cuvettes were initially saturated from the bottom with tap water so that the volumetric water content was *θ* = 0.36 cm^3^/cm^3^ and closed with Parafilm. First, the FID was measured using a single pulse scan. We inserted two 5 mm NMR tubes as markers into this sample, which were filled with 0.04 and 0.1 M CuSO_4_ in a 33% water/67% D_2_O mixture. Next, the bundle was scanned with a multislice multiecho pulse sequence (MSME) to determine *T*_2_ using following parameters: echo time *t*_E_ = 1.6 ms, representing the shortest possible value of the multislice imaging sequence for these systems, number of echoes *n*_E_ = 32, 20 slices with a thickness of 2 mm, and a gap of 0.1 mm. The receiver bandwidth was 300 kHz and 16 scans were averaged using a repetition time of 1.0 s. Carr–Purcell–Meiboom–Gill (CPMG) curves were obtained by plotting the average intensities in a ROI inside one column using Fiji. 

To determine *T*_1_, we used a single echo multislice sequence with the same settings as described above with the only exception that only one echo was monitored. We set 18 different inversion times in the range between 4.2 ms and 300 ms, plus one reference scan without inversion–recovery (IR) preparation. The *T*_1_ relaxation curve was constructed by normalization of the IR prepared scans on the reference scan in a selected ROI. Finally, the relaxation spectra were obtained by inverse Laplace transformation of the time domain data using Prospa (Magritek, Wellington, New Zealand). 

Finally, sample S1 was scanned with ZTE to compare the ZTE images to the MSME images using following parameters: FoV 70 × 70 × 90 mm^3^, excitation bandwidth 1.2 MHz at a block pulse length of 1 μs for a flip angle of 2.5°. The receiver bandwidth of 300 kHz and the number of 64 points per spoke in radial ***k***-space resulted in an acquisition time of 0.213 ms. The number of projections was 51897. Repetition time was *t*_R_ = 6 ms, and 8 scans were averaged.

### 4.3. Sample S2

Sample S2 served as test phantom for the comparison of different pulse sequences at the application lab of Bruker. We filled medium sand FH31, sandy loam, and silt loam into a 20 mm wide cuvette plus a 5 mm NMR marker tube filled with 25% water/D_2_O mixture, see Table 1. The sample was scanned in saturated state by UTE and ZTE pulse sequences with different ratios of excitation and receiver bandwidths to demonstrate the different extend of *T*_2_* blurring artefacts.

### 4.4. Sample S3

**Figure 8:**
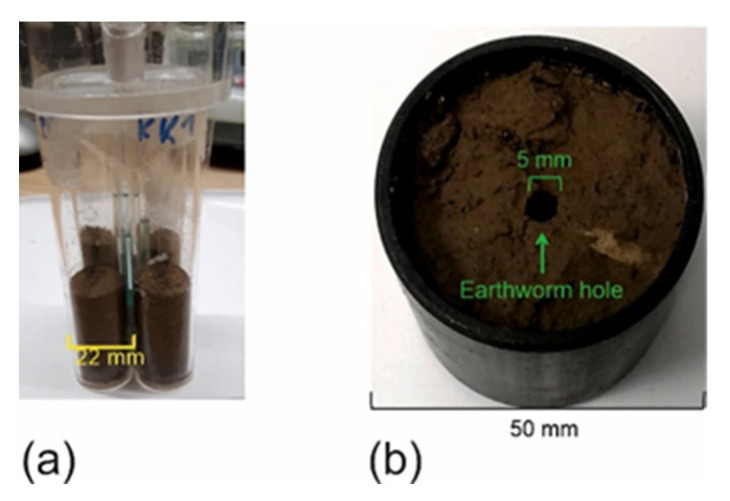
Test samples and soil core. (**a**) Sample S3 consisting of four 22 mm × 100 mm cuvettes filled with sandy loam soil and three reference standard 5 mm NMR tubes. (**b**) Sample S4: Soil core from Selhausen test site in a 100 cm^3^ PVC cutting cylinder (silt-loam).

To determine the MRI images of different water contents, we assembled a new sample analogous to sample S1 but with three additional 5 mm NMR reference tubes filled with 10%, 20%, and 30% water/D_2_O mixtures and 0.2 M CuSO_4_ (Table 1, Figure 8a). Water was sucked out of three cuvettes to adjust volumetric water contents of *θ* = 0.10, 0.19, 0.28, and 0.40 cm^3^/cm^3^. These data were obtained by normalization of the mass differences between dry and wet soil cuvettes on the bulk volume of the packed soil. This sample was scanned with ZTE using following parameters: FOV 90 × 90 × 90 mm^3^, excitation bandwidth 1.2 MHz at a block pulse length of 1 μs for a flip angle of 3°. The receiver bandwidth of 300 kHz and the number of 64 points per spoke in radial ***k***-space resulted in an acquisition time of 0.213 ms. The number of projections was 51897. The repetition time was *t*_R_ = 6 ms and 4 scans were averaged so that the total measuring time for one scan was 20 min 45 s.

### 4.5. Sample S4

A natural soil core, sample S4, was taken from the topsoil at 5 cm depth in the lower part of the test site Niederzier-Selhausen, Germany (Figure 8b). The texture is silt loam with few inclusions of gravel and stones (Table 1). The cutting cylinder was produced from PVC and had an inner diameter of 50 mm and a total volume of 100 cm^3^ (Figure 3b). It was closed at the bottom by a filter plate so that the core could be saturated gently with water from bottom without disturbing the macropore structure. After placing it into the scanner, it was irrigated from top at different rates and the percolating water was collected below outside the magnet. 

Scan parameters of the ZTE sequence were a field of view of 70 × 70 × 90 mm^3^. The excitation bandwidth was 1.2 MHz at a block pulse length of 1 μs for a flip angle of 3°, receiver bandwidth of 300 kHz resulting in an acquisition time of 0.213 ms. The number of projections (spokes of 64 points length in radial ***k***-space) was 51897. Repetition time was *t*_R_ = 2 ms for 1 scan so that the total measuring time for one scan was 1 min 43 s We recorded 35 individual scans during irrigation periods of 1 h to monitor the infiltration and desiccation processes with high temporal resolution. The data were reconstructed with Paravision and further data processing and displayed with Matlab (The Mathworks Inc., Natick, MA, USA) and Fiji [33].

### 4.6. Sample R1: Homogeneity Phantom

To compensate for radial inhomogeneities of the ZTE and UTE sequences resulting from inhomogeneous *rf* irradiation and the reconstruction process we set up a phantom consisting of 55 mm internal diameter Teflon cylinder, filled with 0.1 M CuSO_4_ in 75%/25% D_2_O/H_2_O mixture to a height of 53 mm. This was scanned with identical parameters as the soil samples. The raw images obtained with ZTE and UTE were normalized on this homogeneity phantom.

## 5. Conclusions

We have shown that quantitative 3D imaging of water content in natural soil samples is possible by MRI-ZTE even for unsaturated soil materials. The obtained resolution in the range of 0.5 mm was controlled by the size of the FOV divided by the number of points, typically 128. This results in a minimum total measurement time of 7 min for a repetition time of 8 ms, sufficient for monitoring rapid infiltration processes. The length of the FID decay may limit the effective resolution so that one should keep the acquisition time as short as possible by adjusting large acquisition bandwidths. This in turn requires parallel high excitation bandwidths on the order of 1 MHz to excite the entire sample homogeneously in presence of the space-encoding gradients, which are already switched on at the time of the excitation pulse. For the image processing normalization to the image of a homogeneity phantom or the creation of difference-images are advantageous to compensate for inherent inhomogeneities of the rf-field and intensity scattering over the entire FOV by solidlike ^1^H containing material. With these requirements taken into account, the method is a valuable instrument for monitoring changes in water content patterns in natural soil cores by infiltration, desiccation or root-soil processes.

## Figures and Tables

**Table 1:** Description of the samples.

Sample	Type	Texture (Weight-%)			Porosity (cm^3^/cm^3^)
		Sand	Silt	Clay	
S1	Kaldenkirchen sandy loam	73	23	4	0.4
S2	Selhausen silt loam	13	70	17	0.45
	Kaldenkirchen sandy loam	73	23	4	0.4
	FH31, sand	100	0	0	0.36
S3	Kaldenkirchen sandy loam	73	23	4	0.4
S4	Selhausen soil core, A-horizon	13	70	17	0.55
R1	0.1 M CuSO_4_ in 75%/25% D_2_O/H_2_O	-	-	-	-

## Data Availability

Not applicable.

## Sample Availability

Samples of the compounds described in Table 1 are available from the authors.
